# A Novel Approach to Research Synthesis with the Distillation and Matching Model: Application to the Prevention of Youth Social, Emotional, and Behavioral Problems

**DOI:** 10.1007/s11121-025-01766-2

**Published:** 2025-01-08

**Authors:** Lauren E. Oddo, Bryce D. McLeod, Kevin S. Sutherland, Jason C. Chow, Jennifer R. Ledford, Grace W. Li

**Affiliations:** 1 Department of Psychology, Virginia Commonwealth University, 806 West Franklin Street, PO Box 842018, Richmond, VA 23284-2018 USA; 2 School of Education, Virginia Commonwealth University, Richmond, USA; 3 Department of Special Education, Vanderbilt University, Nashville, USA

**Keywords:** Distillation and Matching Model, Social-emotional functioning, Elementary school

## Abstract

It is difficult for consumers to access the evidence base for prevention programs to determine which models or practices have the strongest empirical support for improving youth social, emotional, and behavioral (SEB) outcomes within their specific service contexts. Researchers can address this evidence-to-practice gap through innovations in research synthesis. The Distillation and Matching Model (Chorpita et al., 2005), an approach to research synthesis developed for the mental health field, is designed to identify what works for whom and under what conditions via three steps. In this paper, we describe the Distillation and Matching Model and suggest that applying this approach to the prevention literature for youth SEB problems may help bridge the evidence-to-practice gap. The first step, distillation, involves identifying “practice elements,” defined as the goal or general principle guiding a discrete practice (e.g., praise) targeting a specific domain of SEB outcomes. This step produces a standard set of terms for the individual practices used across the literature that are studied in isolation and comprise comprehensive intervention models. The second step involves identifying “common elements,” or the practice elements found in studies that meet standards of methodological rigor and report significant improvements in youth SEB outcomes. The third step, “matching,” is a method for matching common element profiles (combinations of common elements) to intervention and personal characteristics to identify what combinations of common elements work for whom and under what conditions. The Distillation and Matching Model can provide a method for researchers to generate actionable information about common elements that can be used to develop and evaluate tailored interventions.

There has been a proliferation of research to develop interventions and practices to prevent or ameliorate youth social, emotional, and behavioral (SEB) problems. This work has primarily focused on developing evidence-based practices (i.e., a specific behavior or action, such as praise, e.g., Sutherland et al., 2000) or comprehensive models (i.e., manualized, pre-packaged programs or protocols containing multiple discrete components or practices such as First Step to Success, e.g., Walker et al., 1998) that effectively target SEB outcomes. Despite this growing literature, there remains an evidence-to-practice gap: Evidence-based prevention programs are not routinely implemented and sustained in the community contexts where youth typically access care.

One factor contributing to the evidence-to-practice gap is that consumers can find it challenging to access and use evidence to decide how best to meet the specific needs of the populations they serve (Chambers et al., 2013; Odom, 2009). This highlights a problem with knowledge translation or the process by which knowledge generated by research (i.e., evidence) is moved into the hands of those who wish to select evidence-based programs to implement in community settings (i.e., practice; Canadian Institutes of Health Research, 2017; Graham et al., 2006). Individual studies and literature reviews are primarily written for scientists rather than practitioners, consumers, or other interested parties. Consequently, there is a gap in the translation of knowledge between the production of research and the ability of consumers to access the evidence and apply findings in everyday practice.

One way to help close the evidence-to-practice gap is to use novel approaches to research synthesis that make information about evidence-based models and practices more accessible and relevant by identifying what works for whom and under what conditions (Chorpita et al., 2011; Graham et al., 2006). The Distillation and Matching Model, initially developed in the mental health field, is an approach to research synthesis designed for this purpose (Chorpita et al., 2005, 2011). In this paper, we propose that applying the Distillation and Matching Model to the education literature will create two innovations. First, applying the model will make findings more accessible by producing a standard set of terms to describe the practices used in the literature to address SEB outcomes. To date, efforts to identify evidence-based models and practices have used various levels of analysis—e.g., organized conclusions at the model level (e.g., what models promote SEB outcomes?) or the practice level (e.g., what discrete practices promote SEB outcomes?)—which has made comparing findings across literature reviews and meta-analyses difficult (Chorpita et al., 2005; Colquhoun et al., 2014). Generating a standard set of terms paves the way for a second innovation—making findings more relevant by using all studies to identify what works for whom and under what conditions. The prevention literature comprises studies that employ a variety of causal designs (e.g., single-case (SCD), quasi-experimental, randomized controlled trials (RCTs)) to evaluate what models and practices work to improve SEB outcomes (Shadish et al., 2015). However, most research syntheses do not include studies that utilize different designs, which limits accessibility (i.e., a single review does not include all studies) and relevance (i.e., conclusions about what works are not based on all studies) of reviews (Shadish et al., 2015). Applying the Distillation and Matching Model to the education literature may thus help to close the evidence-to-practice gap by allowing conclusions about what works for whom and under what conditions to be based on the existing evidence.

## The Evidence-to-Practice Gap in the Education Sector

Much of the evidence-based programming for youth SEB problems in elementary schools fails to reach scale (Lyon & Bruns, 2019). This is unfortunate because approximately one in five elementary children display SEB problems, such as poor peer relationships, internalizing problems, or challenging behaviors (Ringeisen et al., 2020). In turn, SEB problems can impede a child’s ability to fully benefit from educational experiences and cultivate the necessary academic skills for school success (Reinke et al., 2023). Even among those without preexisting academic skills deficits, the presence of SEB problems significantly increases the risk of long-term academic impairments (O’Conner et al., 2011; Spilt et al., 2012), contributing to poor educational, occupational, and mental health outcomes (Polanczyk et al., 2015; Ringeisen et al., 2020). Given the ubiquity of SEB problems in schools and the negative impact of SEB problems on vital developmental outcomes, implementing evidence-based practices and models in schools may help improve SEB functioning (Greenberg & Abenavoli, 2017; Lyon & Bruns, 2019).

Elementary schools regularly receive pressure from policy and educational agencies to adopt classroom-based evidence-based models and practices (e.g., Every Student Succeeds Act, 20 U.S.C. § 6301., 2015). However, researchers and administrators struggle to access the evidence base to identify the models and practices that fit the SEB outcomes within specific school contexts (Chorpita et al., 2011; Cook & Odom, 2013). Once an evidence-based model is selected, school systems can struggle to deliver the model with fidelity, often due to a poor fit between the model and the school context (Locke et al., 2015, 2019). Because evidence-based models and practices do not scale easily to elementary schools, the return on investment for their development and evaluation has been low, considering the millions of children who experience SEB problems and have yet to benefit from evidence-based models and practices (McLeod et al., 2020).

Identifying what works for whom and under what conditions can address barriers to selecting evidence-based models and practices in specific schools (Chorpita et al., 2011; What Works Clearinghouse, n.d.). Models and practices that promote positive SEB outcomes do not demonstrate the same efficacy across all youth or school contexts (Conroy et al., 2018; Durlak et al., 2011). Successful implementation thus depends, in part, on the ability to select a model or practice that has the strongest evidence for youth in a particular context—i.e., interventions that can be tailored to the SEB problems experienced by children in a specific school (Chen et al., 2021; Farmer et al., 2016). Tailoring is consistent with research indicating that adapting evidence-based models to fit specific contexts improves uptake and implementation (Chow & Hampton, 2023; Marques et al., 2019). However, to develop tailored interventions, research synthesis must first identify what works for whom and under what conditions.

It is essential to base conclusions on all relevant studies to identify what works for whom and under what conditions. Meta-analytic reviews focused on models or practices’ effect on elementary students’ SEB outcomes have provided valuable contributions, though these reviews have typically organized findings at the model (Durlak et al., 2011; Taylor et al., 2017) or practice (Barton et al., 2017) level. The education literature has a rich tradition of using SCD and group designs to evaluate practices and models (see Sutherland et al., 2019). However, meta-analyses do not typically include studies utilizing both because the designs are conceptually and procedurally distinct, and there is yet to be consensus on a method for combining effect sizes across the designs (Shadish et al., 2015). When findings from research synthesis are organized at different levels of analysis, it can be challenging for consumers to use the information to identify which model or practice is the best fit for a specific context because the information comes from a subset of the literature (Chorpita & Daleiden, 2009; Powell & Dunlap, 2009). An alternative approach is to adopt a standard level of analysis that can be applied across practices, manuals, models, and studies (Chorpita et al., 2005; Institute of Medicine, 2015). Adopting a standard level of analysis has the potential to enhance the impact of research synthesis focused on prevention programs for children with SEB problems in elementary schools by (a) identifying what practices comprise the models, (b) providing a way to compare findings from studies that focus on models and practices, and (c) basing conclusions on all available literature. A standard level of analysis could thus help identify what works for whom and under what conditions.

## Distillation and Matching Model: A Novel Approach to Research Synthesis in Education

**Fig. 1:**
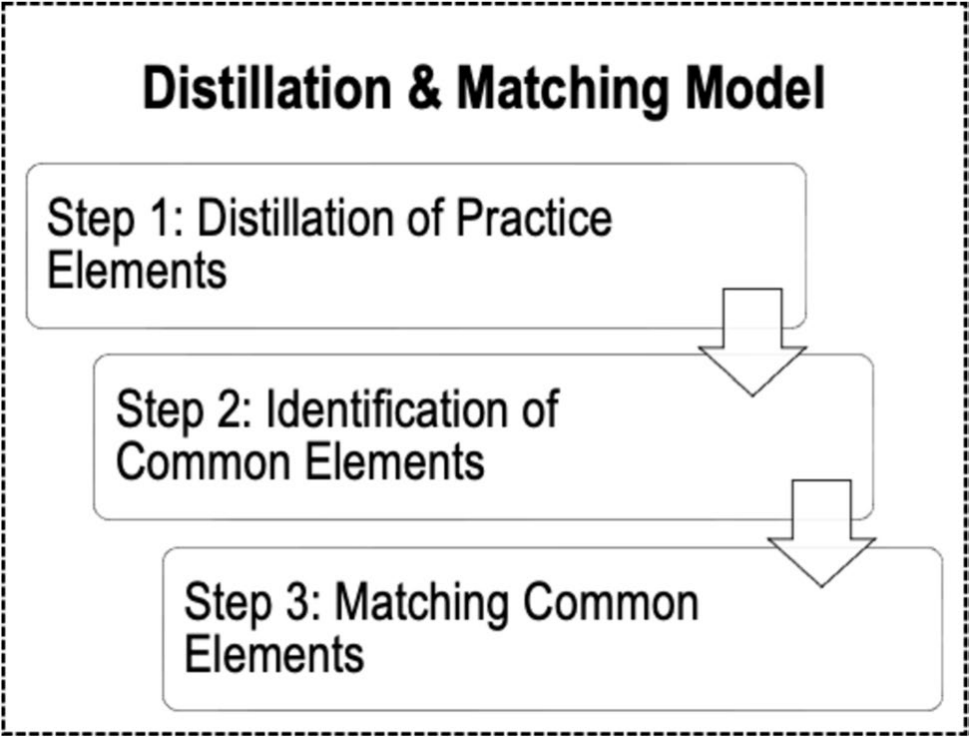
Distillation and Matching Model three-step process

The Distillation and Matching Model is an approach to research synthesis intended to identify what works, for whom, and under what conditions (Chorpita et al., 2011). The Distillation and Matching Model originated in the mental health field to address inefficiencies in translating evidence to practice that resulted from synthesizing the evidence at the level of comprehensive models (Chorpita et al., 2005)—i.e., an over-emphasis on evaluating evidence for specific protocols rather than the strategies that characterize successful interventions. As illustrated in Fig. 1, the Distillation and Matching Model articulates the steps to synthesizing the evidence that can identify what works, for whom, and under what conditions. Applied to prevention programs in education, this approach to research synthesis would provide innovations that can advance efforts to span the evidence-to-practice gap.

### Step 1: Distillation of Practice Elements

**Fig. 2:**
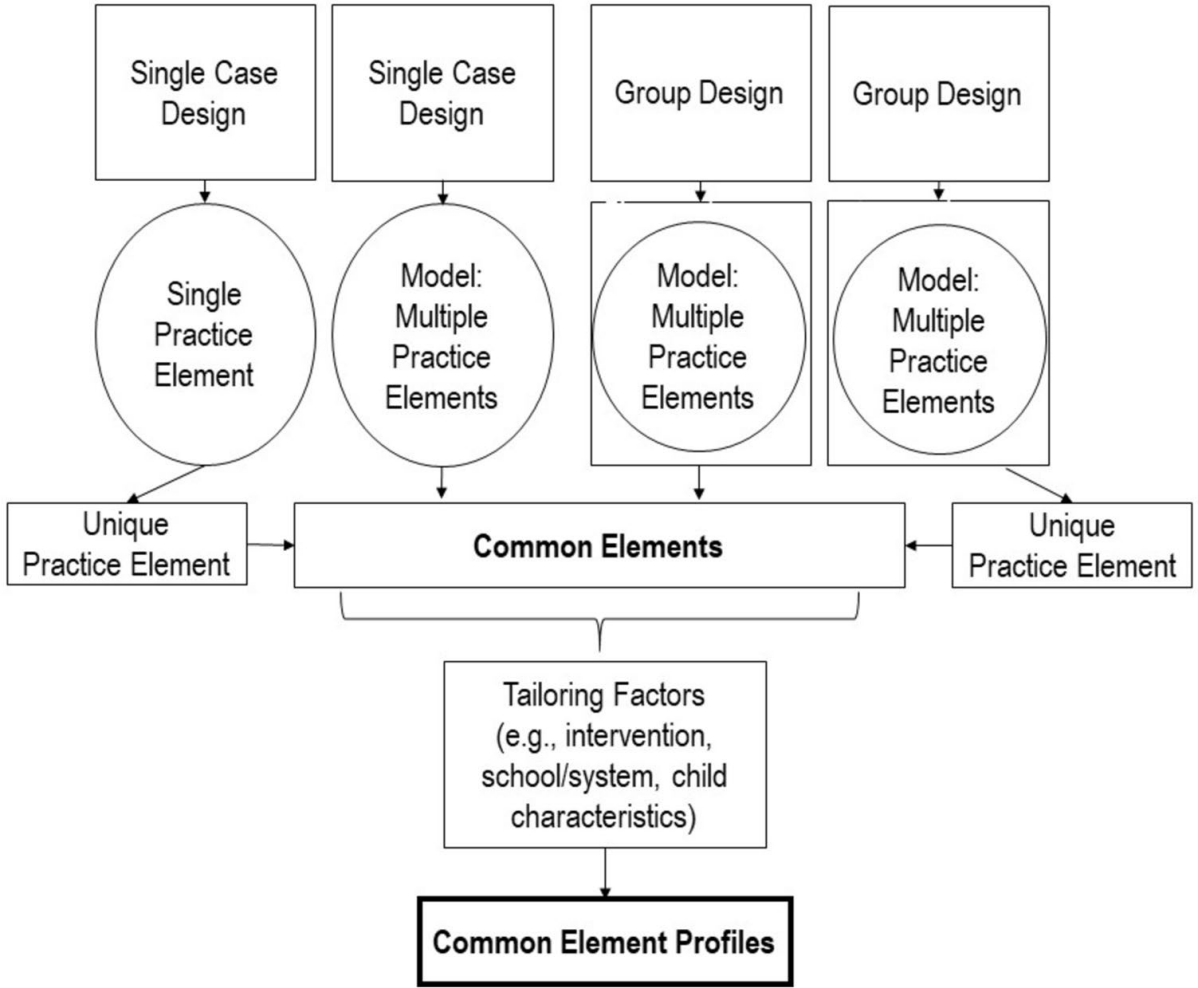
Schematic of synthesis and tailoring across designs to create element profiles

The Distillation and Matching Model first uses a distillation process, which involves researchers identifying the “practice elements” found across multiple models and practices evaluated in studies that meet standards of methodological rigor (Fig. 1; Chorpita et al., 2005). Practice elements, also called “therapeutic strategies” (i.e., general principle that guides an intervention; Beutler & Baker, 1998) and “evidence-based kernels” (i.e., units of behavior influence that cannot be reduced further without losing their function; Embry & Biglan, 2008), are defined as the goal or general principle that guides a practice (e.g., *praise*; Sutherland et al., 2019) that targets an SEB outcome (McLeod et al., 2017). Importantly, practice elements are discrete and can be studied in isolation or as part of a larger model (Bernstein et al., 2015; Chorpita & Daleiden, 2009). The goal of distillation is for researchers to define the practice elements found across multiple practices, manuals, models, and studies in a particular literature (Chorpita et al., 2005; Institute of Medicine, 2015; Taylor et al., 2017). This provides a standard level of analysis that produces information on the practice elements found in the literature, which can be applied across designs not typically included in the same review (e.g., SCDs; Fig. 2; Shadish et al., 2015), thus enabling conclusions based on all available data in its various forms.

Several methods have been used to distill practice elements. Some start with a codebook of pre-defined practice elements used to code for practice elements across a literature (Chorpita & Daleiden, 2009; Chorpita et al., 2005). In this approach, the list of practice elements can be developed via input from experts and relevant parties (Chorpita & Daleiden, 2009; Chorpita et al., 2005) or a review of treatment fidelity measures (Hogue et al., 2019). Other methods do not start with an explicit set of practice elements and instead work to extract practice elements from the literature. Often, this approach utilizes iterative steps to distill practices from individual manuals or studies and combine them into practice elements (e.g., Engell et al., 2023; Garland et al., 2008). Each method has pros and cons; the pre-defined approach may be faster, whereas the second approach may identify a wider range of practice elements (McLeod et al., 2017).

The distillation of practice elements produces a key outcome, a standard set of terms for the practices studied in the literature. This represents an essential first step in identifying what works for whom and under what conditions (Colquhoun et al., 2014; Morgan et al., 2018). Many areas of prevention research in schools have yet to adopt a standard set of terms for the practices studied in the literature (Embry & Biglan, 2008; Sutherland et al., 2019). Instead, various terms are used for the same practices contained in models, even though models often include shared practices (e.g., token economy, merit system, earn-and-exchange system, and reward system; see McLeod et al., 2017; Sutherland et al., 2019). Inconsistent terminology across various models makes it difficult for researchers to compare findings across studies and to communicate research findings to consumers (Colquhoun et al., 2014). Moreover, this has resulted in a problem of empirical redundancy, with Okamura et al. (2020) finding a marked increase in the number of distinct intervention models for youth disruptive behavior problems in the past 40 years (i.e., 131). However, the number of practice elements in the literature (i.e., 48) has remained unchanged since the 1990s. The growing number of intervention models combined with the inconsistent terminology makes it difficult to compare findings across studies and communicate findings between researchers and consumers (Colquhoun et al., 2014). Research synthesis must, therefore, produce consistent terms for the practices studied in the literature before identifying what works for whom and under what conditions (Colquhoun et al., 2014; Institute of Medicine, 2015). Step 1 of the Distillation and Matching Model accomplishes this goal.

### Step 2: Identification of Common Elements

In step 2, researchers identify “common elements,” which are the practice elements (e.g., praise; Engell et al., 2023) found across multiple studies that meet standards of methodological rigor and report significant positive effects based on the criteria used to define evidence-based practice (e.g., APA Presidential Task Force on Evidence-Based Practice, 2006). Whereas identifying practice elements leads to a universal set of terms to communicate findings, distilling common elements determines which are evidence-based (i.e., what works; Chorpita & Daleiden, 2009; Institute of Medicine, 2015). Various approaches have been used to identify common elements. Some have identified common elements by only distilling practice elements from programs that demonstrate significant positive effects (Garland et al., 2010; Hogue et al., 2019). More commonly, groups utilize vote-counting procedures to identify common elements (Chorpita et al., 2005, 2007; Leijten et al., 2019). A few vote-counting approaches exist. One determines the frequency of a specific practice element associated with “winning” studies (i.e., studies that meet methodological standards and demonstrate significant effects) across the literature for a particular SEB outcome. This approach provides a single winning or non-winning “vote” per study for the primary outcome (Chorpita et al., 2011). Another approach is to use effect sizes generated via meta-analytic methods to identify common elements (Leijten et al., 2019). Effect sizes meeting criteria (e.g., above a pre-specified value) are considered a “win.” Researchers using this approach can generate a single effect size per study (e.g., for the primary outcome) or a separate effect size for each SEB domain assessed within a study.

When selecting a method to identify common elements, it is critical to consider the benefits and drawbacks of each method. Considerations include the time required to conduct a review and the characteristics of the literature. Vote-counting procedures typically take longer than focusing on evidence-based programs (i.e., programs that demonstrate significant effects) because they require researchers to review more studies. Frequency counts may be a good choice for vote-counting procedures if the literature is characterized by similar designs (e.g., primarily use RCTs; Chorpita et al., 2011). If, on the other hand, the literature includes different designs (e.g., group, quasi-experiment, SCD) or generates different effects (e.g., across SEB domains; Tanner-Smith et al., 2018), an effect size approach may offer advantages. This approach allows researchers to adjust the threshold for a “win” across student or intervention characteristics (e.g., SEB domains, intervention tiers, group vs. SCD design) and thus pool votes across the literature.

Once votes are assigned, researchers identify common elements. Guided by definitions of evidence-based practice, researchers often require practice elements to occur in a minimum number of studies (replicability) to be included in step 2 (Chorpita et al., 2011). The practice elements are then sorted in descending order (most to least winning votes), and the practice elements that receive the most winning votes are considered a common element for each SEB domain (see Chorpita et al., 2005; Leijten et al., 2019). Additional steps can be taken before defining the common elements. For example, it is possible to consider the ratio of winning to non-winning votes for each practice element when defining common elements, as this determines the strength of support for that practice element across the literature. As is evident by this description, the threshold used to identify common elements can be modified (e.g., change the vote-counting procedure or the effect size magnitude used) so researchers and relevant parties can define the level of evidence they want to use to identify common elements. This is particularly useful when applying these methods across a range of contexts and purposes. With that said, the end product of step 2 is a list of common elements for each SEB domain.

### Step 3: Matching Common Elements

By step 3, the Distillation and Matching Model produces common elements, or the practice elements determined to be evidence-based. The final step in the Distillation and Matching Model involves matching combinations of common elements, called “common element profiles,” to critical factors like intervention and child characteristics (Bernstein et al., 2015; Boustani et al., 2020; Chorpita & Daleiden, 2009). The third step rounds out the Distillation and Matching process since identifying common elements alone (i.e., step 2) does not yield clear implications about when, where, and how to incorporate the various common elements into practice (Colquhoun et al., 2014). This last step determines what common element profiles map onto specific intervention (e.g., universal, indicated, tertiary prevention tiers) and student (e.g., race/ethnicity, problem type, age, gender) characteristics.

These analyses can thus answer questions related to what works and for whom, such as do common element profiles vary across (a) intervention tiers (e.g., universal, secondary), (b) SEB problem types, and (c) student characteristics within SEB problem types (e.g., are distinct common element profiles found across characteristics). At the practitioner level, this information could indicate the combination of common elements to be delivered as a Tier 2 intervention for a 5th-grade female student presenting with unwanted problem behavior (Chorpita et al., 2014). At the school level, an administrator could determine what common element profile best fits the most common SEB problems in their school, given the demographic characteristics of the student body (Bernstein et al., 2015; Boustani et al., 2020; Chorpita & Daleiden, 2009). Common element profiles can thus inform care coordination at the teacher or school level (Chorpita et al., 2008, 2011). It is important to note that it is possible to match additional factors to common element profiles, such as school type (e.g., public, private), context (e.g., state politics, teacher-student ratio), and geography (e.g., rural, suburban, urban). The only limitation to what characteristics can be examined is whether enough studies report the relevant information.

## Addressing the Evidence-to-Practice Gap by Optimizing Knowledge Translation

**Fig. 3:**
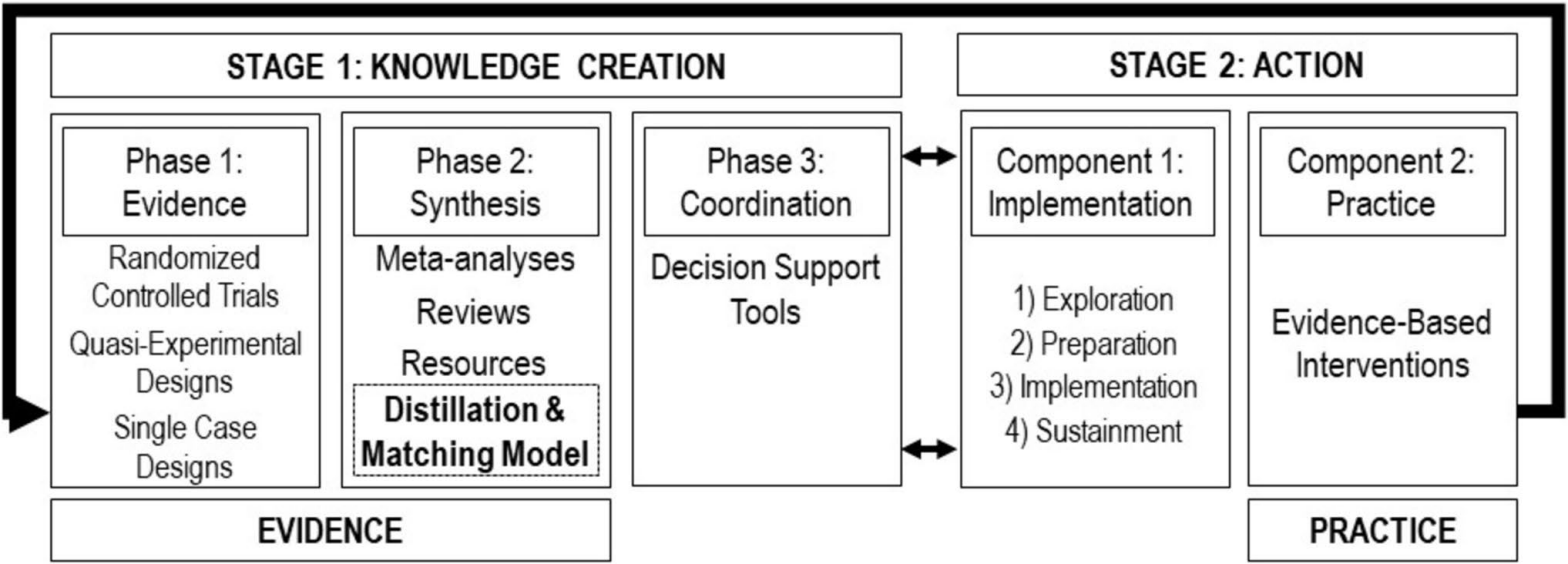
Knowledge Translation Model

Situating the Distillation and Matching Model within a Knowledge Translation Model can help illustrate how this novel approach to research synthesis can help span the evidence-to-practice gap within the education literature. As depicted in Fig. 3, the Knowledge Translation Model explains a two-staged process to move research into the hands of people in practice (e.g., teachers and school administrators): (a) knowledge creation and (b) action (Chorpita et al., 2011; Graham et al., 2006). On the model’s left side, knowledge creation is the production and refinement of evidence, consisting of three phases: (a) evidence, (b) synthesis, and (c) coordination. Researchers use experimental designs, such as RCTs, quasi-experimental designs, and SCDs, in the evidence phase to generate research reports regarding specific models or practices (Graham et al., 2006). These data are considered “raw evidence,” which are variable in quality, often written for a scientific audience, and frequently difficult for consumers to interpret and use (Chorpita et al., 2011). Therefore, the next phase in knowledge creation is synthesis, where researchers summarize the raw evidence by organizing the multitude of studies and data into meta-analyses, systematic reviews, and resources (e.g., APA Division 53, Cochrane Collaborative, CASEL, What Works Clearinghouse). Upon synthesis, coordination involves researchers translating the evidence into user-friendly decision-support tools that directly help consumers (e.g., administrators and teachers) access the evidence to determine how best to address specific outcomes within their school or classroom context.

Action is the second stage depicted in the Knowledge Translation Model, which involves applying scientific evidence to particular school contexts. This phase includes implementation and practice components. The implementation component is “the scientific study of methods to promote the systematic uptake of research findings and other evidence-based practices into routine practice” (Eccles & Mittman, 2006, p. 1). Successful implementation involves a four-part sequence (Aarons et al., 2011; McLeod et al., 2020): (a) exploration, (b) preparation, (c) implementation, and (d) sustainment. In exploration, consumers consider the various evidence-based models or practices to identify a program or practices that can improve an identified SEB outcome in a particular school. Preparation entails selecting an evidence-based model and planning how to integrate it into a specific school context (i.e., identifying what implementation supports are needed). For example, this step would identify what training teachers need and how fidelity monitoring would be incorporated into regular practice. In implementation, the selected evidence-based model or practice is delivered in a school or other service context. Sustainment represents the long-term integration of an evidence-based model or practice into routine care. Finally, the practice component signifies the last part of the action phase, which generates the evidence-informed practices that result from applying scientific evidence to a given population and context. Ultimately, the process ends with interventions tailored to the specific SEB problems of children in a school (e.g., unwanted behaviors).

If the flow of knowledge creation to action is highly efficient, then consumers would be able to use decision-support tools to assess the research evidence, select an evidence-based model or practice that fits their needs, and implement the model with the best implementation supports to ensure the model or practice is effective and sustained (Chorpita & Daleiden, 2009; Wexler et al., 2023). However, the pathway between knowledge creation and action for preventing youth SEB is not optimized, primarily because few decision-support tools exist. To address this problem, researchers must develop practical decision-support tools designed specifically for end-users (Sterrett et al., 2020). To achieve this goal, researchers must first make the products of research synthesis more accessible and relevant to consumers. As illustrated above, the Distillation and Matching Model can achieve this goal by creating a standard set of terms to communicate to consumers and identifying what works, for whom, and under what conditions. Together, this information represents the necessary ingredients to develop practical decision-support tools to help span the evidence-to-practice gap.

## Putting the Steps Together: An Example from the Mental Health Literature

Chorpita and Daleiden (2009) provide a template from the mental health literature for applying the Distillation and Matching Model to education. Specifically, they created a list of practice elements based on expert judgment and the input of practice partners. The research team then used the list to identify common elements by distilling practice elements from hundreds of RCTs that demonstrated significant positive effects in the youth mental health literature (Chorpita & Daleiden, 2009; Chorpita et al., 2005). Upon deriving the common elements, the researchers mapped child characteristics (i.e., problem type, age, gender, race/ethnicity) onto common element profiles (Chorpita et al., 2011). With this information, the researchers developed a novel decision-support tool called Managing and Adapting Practice (MAP; Chorpita et al., 2014). Clinicians are now able to use MAP to select and implement common elements. Clinicians enter information about their clients into MAP, and the system determines what common elements have the most robust empirical support given the child characteristics (e.g., problem type, race/ethnicity, age; Buckingham et al., 2019; Southam-Gerow et al., 2014). The data that drives MAP has also been extended to tailor practices to various service sectors, such as mental health systems and wraparound services. This allows administrators to determine what common element profiles have the strongest empirical support for the specific youth characteristics represented in a particular sector of mental health care (Boustani et al., 2020; Chorpita et al., 2011), which has important implications for workforce training. Upon evaluation, tailored interventions comprised of common element profiles produced with data from MAP have been shown to outperform traditional “non-tailored” interventions in RCTs (Chorpita et al., 2014, 2017; Weisz et al., 2012) and significantly increased the use of common elements within sizeable mental health systems (Higa-McMillan et al., 2020; Southam-Gerow et al., 2014).

## Distillation and Matching Model to Address Translational Problems in Schools

The work from mental health indicates that the Distillation and Matching Model has the potential to generate data to develop decision-support tools that can be used to guide practice and inform care for a given service sector. Despite its promise, no known work has applied this model to synthesize the existing evidence in education across multiple experimental designs—resulting in a lack of representative evidence. Taking lessons from the youth mental health field has potential benefits for applying the Distillation and Matching Model to preventing youth SEB problems in schools. Indeed, some have called for using practice elements or evidence-based kernels in the prevention literature, including in the education sector (Embry & Biglan, 2008; Sutherland et al., 2019). However, this work has not progressed to the point where it has identified what works, for whom, and under what conditions, and thus cannot be used to inform the development of decision-support tools to help schools identify and apply research evidence.

We propose several key strategies to advance the Distillation and Matching Model and apply it to education literature, aiming to bridge the gap between evidence and practice. Information derived from the Distillation and Matching Model could support schools in identifying and delivering common elements of practices across prevention tiers to meet the needs of their student population best—thereby enhancing equitable service delivery (Chen et al., 2021; Lloyd et al., 2019). Child characteristics, such as race, ethnicity, and gender, influence the generalizability of intervention effects (Farmer et al., 2016; Wehby & Kern, 2014), suggesting that different common element profiles may be needed across the student population. Practices may also not have equal efficacy across children with different racial and ethnic backgrounds—a finding that has been supported by research in the mental health field (Rathod et al., 2018). The practice elements used in schools vary depending on child characteristics such as age; for example, Sutherland et al. (2019) found that group contingency (i.e., applying consequences to a group of children) is frequently used with early elementary students, but this practice was not used with preschoolers in early childhood settings (Korpershoek et al., 2016; McLeod et al., 2017). Moreover, practices can be targeted to specific child concerns; for example, precorrection effectively reduces problem behaviors, while modeling has been shown to improve social skills (Haydon et al., 2017). Future work should seek to apply this model across diverse populations and settings to enhance equitable access to school services.

In applying the Distillation and Matching Model to the prevention of youth SEB problems in schools, it is essential to note that evidence-based practices and models in schools are often organized and delivered via a Multi-Tiered System of Support (MTSS) or Positive Behavioral Interventions and Supports (PBIS) framework (Sugai & Horner, 2009). MTSS and PBIS are based on the public health prevention model and conceptualized within a three-tier continuum of supports (Cook et al., 2010; Sugai et al., 2000). Students receive intensified intervention based on their responsiveness to less intensive support. When unresponsive to Tier 1 (universal) support, students receive more intensive Tier 2 and/or Tier 3 support (Rones & Hoagwood, 2000; Walker et al., 1996). This framework is predicated on the assumption that the appropriate evidence-based support has been delivered with fidelity before intensifying services. Thus, efforts to identify common element profiles via the Distillation and Matching Model process should consider the three-tier prevention framework and aim to understand how these common elements function across different tiers of service delivery (Sugai & Horner, 2009).

Current research using the Distillation and Matching Model also has methodological issues that need additional empirical attention. Thus far, researchers have used various approaches to identify common elements (e.g., applying a codebook with predetermined constructs to the literature, deriving common elements via factor analysis, and using a bottom-up process with consensus coding). Disparate approaches could lead to different conclusions about best practices and models. For example, approaches that include practice elements that are largely predetermined may not capture all practice elements within a given literature (Garland et al., 2008). Thus, some methodologies may produce a more expansive list of elements (e.g., Chorpita et al., 2005 vs. McLeod et al., 2017). Of course, the ideal number of practice elements identified may vary by the purpose of the review.

When creating common element profiles, researchers should avoid simply creating new manualized protocols that require intensive implementation supports (e.g., expensive training and coaching). Involving end-users in each step of the distillation process can help ensure that the Distillation and Matching Process products are designed for ease of use in school settings, helping avoid a mismatch between the design of the common elements and the ability to implement those elements in school settings. Similarly, engaging end-users in selecting common element profiles will help ensure that a tailored intervention is feasible and a good match for the target population. Mixed-method research incorporating the feedback of scientific experts and school partners is likely necessary to achieve these goals. Indeed, enhanced attention to these methodological considerations is needed to support replication efforts and generalizability.

Ultimately, the Distillation and Matching Model has the potential to generate data for researchers to develop decision-support tools for schools to select, adapt, and prepare tailored prevention efforts comprised of common elements. For example, with a decision-support toolkit, a school system may identify what common elements work for their population of interest (Bernstein et al., 2015; Boustani et al., 2020). A toolkit could also provide training and coaching designed to support the quantity (adherence) and quality (competence) of the common elements that comprise the tailored intervention (Bernstein et al., 2015; Boustani et al., 2020). Importantly, developing decision-support tools for schools based on the Distillation and Matching Model requires researchers to consider the support needed for schools to use those tools effectively. Special attention is needed to answer questions about how providers, organizations, or other implementors can learn to reliably apply common elements in their work, using ongoing responsiveness monitoring to calibrate effectiveness. As prevention and intervention efforts are dynamic and should change based on ongoing progress monitoring, decision-support tools must remain flexible and integrate monitoring methods to be maximally effective in the real world.

## Conclusion

Effective knowledge translation depends, in part, on synthesis products that lead to better coordination of evidence and practice. The Distillation and Matching Model is an untapped synthesis technology with clear implications for closing the evidence-to-practice gap across service contexts, including preventing youth SEB problems in schools. Applying the Distillation and Matching Model can generate actionable information about common elements that can be used to develop and evaluate tailored interventions for youth SEB outcomes. Since the Distillation and Matching Model can be used across areas of prevention, we conclude with a few considerations for researchers who may be considering applying the model to new areas.

1. State of the literature—Applying the Distillation and Matching Model requires extensive literature that meets methodological standards and can provide evidence for a specific research question related to intervention or prevention. Thus, it is important to consider if the literature meets the standards for a systematic review (Higgins et al., 2019) to ensure enough studies exist to draw conclusions about effectiveness (Chorpita et al., 2011).
2. Characteristics of the literature—The experimental designs, intervention characteristics, and participant characteristics must be considered, as these features impact decisions at each step in the model. For example, the meta-analytic vote-counting approach may be a better fit for a literature that uses different experimental designs. As another example, knowledge of what client and intervention characteristics are important for tailoring will help determine how to code studies during step 1 in order to prepare for step 3.
3. Goals and resources—The goal of the work must be considered, since it can have implications for the resources required to complete the work. Identifying common elements from an evidence-based model for a specific problem (e.g., parent-training for disruptive behavior problems; Garland et al., 2010) takes fewer resources than a broad review (e.g., mental health treatments for youth internalizing and externalizing problems; Chorpita et al., 2011). In addition, conducting a meta-analysis and distilling the practice elements requires more resources than a vote-counting approach.
4. Identification of common elements—The pros and cons of using different methods to identify common elements are a final factor to consider. Matching the approach to the project’s goal and resources is essential so that the end product is helpful in the field. Other methods not reviewed in this paper provide opportunities to identify common elements. Examples include using network and component network meta-analytic methods designed to isolate components’ direct and indirect effects across a series of multicomponent intervention studies. These methods provide different but complementary pieces of information that may be used to identify common elements.
